# The effect of photobiomodulation therapy associated with casein phosphopeptide‐amorphous calcium phosphate fluoride paste on the treatment of posthome whitening tooth sensitivity and color change: A randomized clinical trial

**DOI:** 10.1002/cre2.817

**Published:** 2024-01-31

**Authors:** Maria E. de O. P. Cardoso, Elma V. Takeuchi, Cristiane de M. Alencar, Raissa A. de Mesquita, Eliane B. Alves, Cecy M. Silva

**Affiliations:** ^1^ Department of Restorative Dentistry, Dental School Federal University of Pará Belém Pará Brazil

**Keywords:** carbamide peroxide, tooth sensitivity, tooth whitening

## Abstract

**Objective:**

This study assessed whether combining photobiomodulation therapy (PBMT) with casein phosphopeptide‐amorphous calcium phosphate fluoride (CPP‐ACPF) paste can effectively reduce post‐home whitening tooth sensitivity (TS) without compromising shade change.

**Methods:**

Fifty participants were selected and assigned to one of four groups: (1) PLACEBO group—received a placebo paste and PBMT simulation; (2) PBMT group—received a placebo paste + PBMT; (3) CPP‐ACPF group—received CPP‐ACPF paste and PBMT simulation; (4) CPP‐ACPF + PBMT group—received both CPP‐ACPF paste and PBMT. The participants used whitening trays containing 22% carbamide peroxide for 2 h a day for 21 days. TS was measured daily using a visual analog scale, while shade change was assessed using a spectrophotometer: before bleaching treatment (T0), after the first (T1), second (T2), and third (T3) weeks of treatment, and 30 days (T4) after completing the whitening treatment.

**Results:**

Intragroup analysis revealed that the PLACEBO group had the highest increase in sensitivity during the whitening treatment. The CPP‐ACPF and PBMT groups showed no significant difference tooth whitening (TW) between weeks regarding aesthetic change. The CPP‐ACPF and PBMT group exhibited a significant reduction in TS between the first and third and between the second and third weeks TW, but not between the first and second. Conversely, the PLACEBO group showed a higher sensitivity than the other groups (*p* < .05). The CPP‐ACPF and PBMT groups did not differ from each other. Furthermore, the CPP‐ACPF and PBMT group showed a greater decrease in sensitivity than the PLACEBO group at T1, T2, and T3 (*p* < .01), and was significantly differed from CPP‐ACPF and PBMT groups only at T2 and T3. All groups confirmed TW effectiveness. Student's and paired t‐test did not reveal any significant difference between groups (*p* > .05).

**Conclusion:**

Therefore, PBMT associated with CPP‐ACPF paste can reduce TS without compromising the efficacy of TW.

## 1 INTRODUCTION

Tooth whitening (TW) uses peroxide‐based oxidizing agents capable that can penetrate the tooth structure and generating reactive oxygen species to oxidize organic pigment molecules (Elfallah & Swain, 2013; Rashid & ElSalhy, 2021). However, because peroxides have low molecular weight, they can also diffuse into interprismatic spaces, and reach the pulp tissue, causing oxidative stress in pulp cells (Benetti et al., 2004; Torres et al., 2010). The released inflammatory mediators released bleaching treatment can stimulate or sensitize nociceptors, leading to tooth sensitivity (TS), one of the most common adverse effects (Elfallah & Swain, 2013; Rashid & ElSalhy, 2021). According to the literature, approximately 51% of patients who subjected to at‐home TW experience this transient sensitivity (Chidchuangchai et al., 2007; Favaro Zeola et al., 2019; Rezende et al., 2016; Splieth & Tachou, 2013).

Although there is no gold‐standard treatment for TS after home whitening, several clinical protocols have been developed to minimize its risk and intensity such as using desensitizing agents and photobiomodulation therapy (PBMT) (Kawamoto & Tsujimoto, 2004; Maran et al., 2018; Moosavi et al., 2016; Rezende et al., 2019; Sasaki et al., 2009). PBMT reduces the release of mediators that exacerbate the inflammatory process caused by bleaching agents by sing a low‐intensity laser. In addition, the analgesic effect of PBMT may also be explained by the reduced nerve conduction velocity, which can alter the patient's pain threshold (Ladalardo et al., 2004; Markowitz & Pashley, 2008a; Wakabayashi et al., 1993). According to a recently published systematic review, PBMT shows promise in preventing postwhitening TS, but further clinical studies are needed to reach a definitive conclusion due to the limited evidence available (Carneiro et al., 2022). Low‐power lasers are classified as red, emitting photons in the visible red range (600–700 nm) and infrared (700–950 nm). The difference lies in the depth of penetration of the different wavelengths, with red light having a penetration capacity of 8–10 mm, while infrared light has a penetration capacity of 2–3 cm. This fact may justify the better performance of infrared lasers in controlling pulp inflammation caused by bleaching (Moosavi et al., 2016).

Desensitizing agents can reduce post‐whitening TS through either a neural mechanism or by limiting the amount of reactive oxygen species that reach the pulp. Casein phosphopeptide‐amorphous calcium phosphate fluoride (CPP‐ACPF), a milk protein complex supplemented with fluorine, has been introduced in the market as a potent remineralizing agent (Guanipa Ortiz et al., 2019). Casein phosphopeptide (CPP) has the ability to binds to and stabilizes soluble amorphous calcium phosphate (ACP). When ACP dissociates into calcium and phosphate ions in saliva, they bind to the tooth surface and form a reservoir with high concentrations of these minerals (Huq et al., 2016). The introduction of fluorine (900 ppm) into this formulation (CPP‐ACPF), enhances its remineralizing effect. By promoting by the deposition of high concentrations of minerals, CPP‐ACPF may help to reduce TS by occluding dentinal tubules and may limit the amount of reactive oxygen species in the pulp (Reynolds et al., 2008).

The likely synergistic action of combining the analgesic and anti‐inflammatory effects of PBMT with the remineralizing potential of CPP‐ACPF paste seems to be a promising therapeutic alternative. However, until now, no clinical studies have evaluated the efficacy of this combination in reducing sensitivity induced by TW. Therefore, this randomized, double‐blind, split‐mouth, placebo‐controlled clinical trial aims to assess the efficay of combining PBMT with CPP‐ACPF paste in reducing TS during home TW without compromising the shade change.

The study tested the following null hypotheses: H01—there is no difference between combining CPP‐ACPF with PBMT and the placebo group in reducing post‐whitening TS; H02—there is no difference in shade between combining CPP‐ACPF with PBMT and the other treatments.

## 2 MATERIALS AND METHODS

### 2.1 Ethical aspects

The present study was designed according to *Consolidated Standards of Reporting Trials—CONSORT* (Schulz et al., 2010) and was submitted to the Human Research Ethics Committee of the Institute of Health Sciences at the Federal University of Pará (*Comitê de Ética em Pesquisa em Seres Humanos do Instituto de Ciências da Saúde da Universidade Federal do Pará* – CEP‐ICS/UFPA). The study was approved under opinion number 49921321.2.0000.0018 and was registered on the ClinicalTrials.gov website under the identifier NCT05298059.

The participants were duly informed about the risks and methods of this study and signed an informed consent form in accordance with the Declaration of Helsinki. All participants were aware that they could withdraw from the study at any time they deemed necessary.

### 2.2 Study site and participant recruitment

This randomized, double‐blind, split‐mouth, placebo‐controlled clinical trial was performed from May to August 2022 at the School of Dentistry, UFPA (Belém, PA, Brazil). Participants were recruited through a research announcement posted on bulletin boards at UFPA.

### 2.3 Sample size

The sample size of this study was defined using the program GPower 3.1 (Heinrich‐Heine‐Universität Düsseldorf, Germany) based on data from a previous pilot study that followed the same method. The calculation adopted an 80% statistical power, a 5% α‐error, and a difference of two units in the visual analog scale (VAS) score, with a 20% sample loss prediction at the end of the study. This resulted in a sample size of 25 participants per group, for a total of 50 participants.

### 2.4 Patient selection process

A total of 50 adult participants, aged from 18 to 30 years, both male and female, with good oral and systemic health and at least 28 teeth, were selected for this study. The participants were required to meet the following inclusion criteria: absence of active carious lesions; sound incisors, canines, and premolars; not presenting with dental hypersensitivity and having A2 or darker teeth. Individuals with periodontal disease, tooth cracks or fractures, restorations and prostheses in anterior teeth, any disease that could cause TS (bruxism, acid reflux, and dentin exposure, among others), and/or severe internal dental darkening were excluded from the trial, as well as smokers, pregnant women, nursing mothers and patients undergoing orthodontic treatment. Individuals undergoing continuous treatment with anti‐inflammatory and/or analgesic drugs were also excluded from this trial.

During selection process, TS was assessed by tactile and evaporative stimulation. The tactile stimulation, which involved cross‐shaped exploratory probe to apply on the buccal surface of the tooth in both the apical‐to‐incisal and mesial‐to‐distal directions. The evaporative stimulus, which was induced by applying an air jet at 40 psi pressure from a triple syringe perpendicularly to the buccal surface of the tooth, 2 mm away from it, for 3 s. Participants' pain response was scored according to VAS. Only individuals who did not experience sensitivity to the stimuli were included in the trial.

### 2.5 Study design

The study participants were divided into two groups (*n* = 25). The right‐ and left‐side quadrants received different treatments (split mouth), thus forming a total of two groups and four subgroups: PLACEBO; PBMT; CPPACPF; CPPACPF + PBMT (Table 1).

**Table 1:** Group division by treatment received in each (right or left) hemi‐arch and treatment description.

Group	Hemi‐arch treatment	Treatment description
GROUP 1 (*n* = 25)	PLACEBO	Application of the paste without the active ingredient, followed by PBMT simulation
	PBMT	Application of the paste without the active ingredient, followed by PBMT
GROUP 2 (*n* = 25)	CPPACPF	Application of the CPP‐ACPF paste, followed by PBMT simulation
	CPPACPF + PBMT	Application of the CPP‐ACPF paste, followed by PBMT

Abbreviations: CPP‐ACPF, casein phosphopeptide‐amorphous calcium phosphate fluoride; PBMT, photobiomodulation therapy.

### 2.6 Randomization

The randomization process was performed in two‐stage approach by an independent researcher who had no involvement in the clinical aspects of the project, using the Bioestat 5.0 software (Sociedade Civil), to generate a computer‐generated randomization table. In the first stage, the participants were randomly assigned to one of two large groups (GROUP 1 and GROUP 2). The second stage involved allocation of the different treatments to the hemi‐arcs (right and left) of each participant, thereby determining the treatment subgroups, which included PLACEBO, PBMT, CPPACPF, and CPPACPF + PBMT.

### 2.7 Blinding

In this double‐blind trial, measures were taken to ensure blinding of both the participant and the evaluator (researcher who performed the statistical analysis) whom did not know the allocation of groups. Operator was not blinded due to the application of laser and its simulation. To prevent differentiation between treatment groups, the paste without the active ingredient (placebo) was mimicked the consistency, flavor and color of the paste with CPP‐ACPF. Both folders were stored in identical containers to prevents the participants from differentiating the pastes. In addition, the sound emitted by the laser during PBMT was simulated positioning a cell phone application, while the tip of the probe was positioned to imitate the sound emitted by the laser during its operation.

### 2.8 Clinical protocol

All participants were provided with a soft toothbrush (CURADENT International AG), toothpaste without desensitizing action and without fluoride, to mitigate possible interferences in the assessments (fluoride‐free toothpaste, My frist‐ Colgate®, Colgate‐Palmolive Company), a tube of 22% carbamide peroxide (FGM; Joinville) and received instructions on how brush their teeth. Before starting the whitening treatment, prophylaxis was performed with a rubber cup (Microdont) mounted on a low‐speed handpiece with pumice paste.

Custom‐made trays for at‐home whitening were prepared by molding the upper and lower arches with alginate (Coltene; Bom Sucesso). A vacuum plasticizer (Bioart) and 1‐mm‐thick Soft‐tray sheets (Bioart) were used to fabricate the whitening trays from the plaster casts. Excess material from the buccal and lingual surfaces was trimmed to 1 mm from the gingival margin. The trays were adjusted in the mouth to ensure a proper fit. Participants were instructed to dispense a drop of the bleaching agent onto each tooth in the tray, up to the second premolars, and to wear the tray 2 h a day, for 21 days.

The desensitizing treatments were administered at four different time points: before whitening (T0) and after one (T1), two (T2), and three (T3) weeks of TW. The treatments were applied on the buccal surface for whitening, including the incisors, canines and premolars.

#### 2.8.1 PLACEBO treatment

The laser simulation was performed for the CPPACPF and PLACEBO groups, so that the laser probe was positioned at three points perpendicular to the long axis of the teeth: two points in contact with buccal surface of tooth (middle third and incisal) and one apical point, in contact with gum, towards the root of the teeth, but not activated. A cell phone application was used to simulate the sound emitted by laser during its activation.

The PBMT and PLACEBO groups received application of the paste without the active ingredient. Placebo paste was applied with a gloved finger, remaining in contact with the tooth surfaces for 5 min. During this period, the participants were instructed to keep their mouths closed without spitting. Then, they spat the entire product (without rinsing) and did not ingest any liquid or solid food for 30 min.

#### 2.8.2 Laser application

The PBMT and CPP‐ACPF groups received the application of low‐intensity laser (Therapy EC, DMC) as follows: laser was applied at three points, perpendicular to the long axis of the teeth. Two points in contact with buccal surface (middle third and incisal) and one point in contact with gum, at apical point towards the root of the teeth. An infrared (IR) low‐intensity laser was used in continuous mode, at 880‐nm wavelength, 100 mW/cm^2^ output power, 1 J energy per spot.

#### 2.8.3 CPP‐ACPF paste application

The paste (GC Corp.) was applied with a gloved finger, remaining in contact with the tooth surfaces for 5 min. During this period, the participants were instructed to keep their mouths closed without spitting. Then, they spat the entire product (without rinsing) and did not ingest any liquid or solid food for 30 min. This protocol was applied to groups CPPACPF and CPPACPF + PBMT.

### 2.9 Tooth sensitivity evaluation

The perception of participant's pain was assessed using a visual analog scale VAS. It is a scale characterized by a horizontal line measuring 10 cm. On this line, there are markings ranging from 0 to 10. Where 0 is located at its left end and 10 at its right end. Zero represents absence of sensitivity, while 10 represents severe tooth sensitivity. Patients were instructed to mark the intensity of their tooth sensitivity each day by drawing a vertical line parallel to the scale's body along the horizontal line. The distance in millimeters from the zero end was measured using a millimeter ruler to determine the patient's level of pain intensity. If participants did not feel any pain they are instructed to circle 0.

The scale was provided to each participant at the start of treatment to monitor pain sensitivity, during the 21‐day whitening period and 30 days following the completion of TW.

### 2.10 Shade determination

The change in tooth shade was analyzed using an electronic assessment method on a Vita Easyshade V digital spectrophotometer (VITA Zahnfabrik). The tooth shade was measured on the middle third of the buccal surface of the maxillary canines. A dense condensation silicone matrix (OPTOSIL‐ Kulzer) was prepared for the upper arch with a round opening 6 mm in diameter, matching the size of the Easyshade tip, towards standardizing the positioning of the device for tooth shade determination.

The color change between baseline and 30 days after the whitening treatment was determined using CIELab (ΔE) and CIEDE 2000 (ΔE00) method. To obtain ΔE, value of L, a* and b* were collected from spectrophotometer. Where L represents lightness and ranges from 0 to 100, with 0 for black and 100 for white. The value of a* ranges from −90 to +70, it represents chromaticity along the red‐green axis (−a* the greenest, +a* the reddest), and b* value ranges from −80 to 100, it represents chromaticity along the yellow‐blue axis (−b* the bluest +b* the yellowest) (Dozić et al., 2003). The collected data were used in the following formula ΔE = [(ΔL*)^2^ + (Δa*)^2^ + (Δb*)^2^]^½^ to obtain ΔE value.

To obtain ΔE 00, the data collected from spectrophotometer were L, H, and C, and it was calculated by the following formula ΔE 00 = [(ΔL∕kLSL)2 + (ΔC∕kCSC)2 + (ΔH∕kHSH)2 + RT(ΔC × ΔHΔHSC)SC × SH)]1∕2, where ΔL, ΔC, and ΔH are the differences in lightness, chroma, and hue, respectively. The SL, SC, and SH weighting functions adjust the total color difference by the variations in L', a', and b' coordinate pairs. Parametric factors k1, kc, and kh were set to 1 for calculations purposes.

### 2.11 Statistical analysis

TS and shade data were tabulated in an Excel spreadsheet (Microsoft Windows, 2010) and analyzed using the open‐source statistical software Jamovi (version 1.8). Normality of the TS data was evaluated using the Shapiro–Wilk test and non‐normality of data distribution was checked (*p* < .05).

The primary outcome of the study was TS reported by the participants. Statistical analysis was performed using the median VAS scores after the first (T1), second (T2), and third (T3) weeks of treatment and 30 days (T4) after completing TW. Intragroup analysis were performed using the Friedman test to compare between time points within each group. Intergroup analysis was conducted using the Wilcoxon and Kruskal‐Wallis tests, for the dependent and independent groups, respectively, to compare the efficacy of different desensitizing treatments.

For the secondary data of variation in shade (ΔE_ab_ e ΔE_00_), the data met the normality criteria, and therefore Student's *t*‐test and paired t‐test were performed for dependent and independent groups, respectively. A significant at a 5% probability level in all tests.

## 3 RESULTS

**Figure 1:**
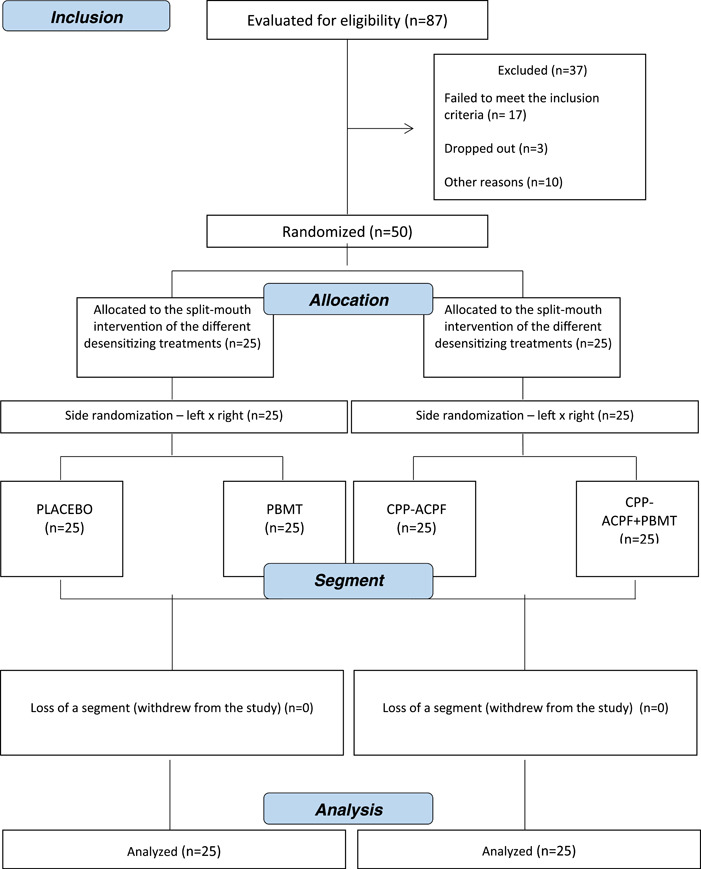
Study design flowchart.

All participants completed all phases of this trial, and their demographic characteristics are outlined in Table 2. The study design flowchart is shown in Figure 1.

**Table 2:** Demographic characteristics of the participants who completed the study and mean and standard variation of age.

	PLACEBO	PBMT	CPPACPF	CPPACPF + PBMT
	(*n* = 25)	(*n* = 25)	(*n* = 25)	(*n* = 25)
Sex *n* (%)				
Female	12 (48%)	12 (48%)	14 (56%)	14 (56%)
Male	13 (52%)	13 (52%)	11 (40%)	11 (40%)
Age				
Mean (±standard deviation)	24.8 (±3.87)	24.8 (±3.87)	24.9 (±4.52)	24.9 (±4.52)

Abbreviations: CPP‐ACPF, casein phosphopeptide‐amorphous calcium phosphate fluoride; PBMT, photobiomodulation therapy.

### 3.1 Tooth sensitivity

The medians and interquartile ranges of post‐whitening TS in the different study periods are described in Table 3. In the intragroup analysis, the PLACEBO group showed a gradual and significant increase in TS during the 3 weeks of the whitening treatment, according to the Friedman test (*p* < .001). The median VAS score was 1 (0–2.5 IQR) in the first week, 2.7 (1–4 IQR) in the second week, and 4.1 (2–4.75 IQR) in the third week. The median TS of the PBMT and CPPACPF subgroups did not significantly differ between weeks (*p* = .247 in the first week, *p* = .384 in the second week, and *p* = .297 in the third week). The CPPACPF + PBMT subgroup showed a significant difference between the median TS between the first and second (*p* = .042) and between the first and third (*p* = .035) weeks, but no significant difference was observed between the second and third weeks (*p* = .787).

**Table 3:** Median and interquartile range of tooth sensitivity in the different study periods.

	Median (interquartile range)				
Groups	Baseline	1st Week	2nd Week	3rd Week	One‐month follow‐up
PLACEBO	0 (0‐0)	1 (0‐2.5)^Aa^	2.7 (1‐4)^Ba^	4.1 (2‐4.75)^Ca^	0 (0‐0)
PBMT	0 (0‐0)	0 (0‐1)^Ab^	1 (0‐2)^Ab^	1.5 (0‐ 3)^Ab^	0 (0‐0)
CPPACPF	0 (0‐0)	0 (0‐1)^Ab^	1 (0‐2.5)^Ab^	1 (0‐2)^Ab^	0 (0‐0)
CPPACPF + PBMT	0 (0‐0)	0 (0‐0)^Ab^	0 (0‐2.36)^Bc^	0 (0‐2.51)^Bc^	0 (0‐0)

*Note*: Different uppercase letters represent significant differences in the intragroup evaluation, for the different tests applied; *p* ≤ .05.

Different lowercase letters represent significant difference in the intergroup evaluation, for the different tests applied; *p* ≤ .05.

Abbreviations: CPP‐ACPF, casein phosphopeptide‐amorphous calcium phosphate fluoride; PBMT, photobiomodulation therapy.

After 1 month of follow‐up, there were no reports of tooth sensitivity by any participant. In the comparison between desensitizing treatments (intergroup analysis), the Kruskal–Wallis and Wilcoxon tests showed that the TS of the PBMT, CPPACPF, and CPPACPF + PBMT groups significantly differed from the TS of the PLACEBO groups in the 3 weeks of treatment (*p* < .001). The PBMT and CPPACPF groups had statistically similar medians (*p* > .05). In the first week, the median TS of the CPPACPF + PBMT group did not significantly differ from the TS of the groups that received PBMT (*p* = .07) and CPP‐ACPF alone (*p* = .299). In the following TW weeks, the CPPACPF + PBMT group showed significantly lower TS values than the PBMT (*p* = .01 and *p* = .005 in the first and second weeks, respectively) and CPPACPF (*p* = .023 and *p* = .01 in the first and second weeks, respectively) groups.

### 3.2 Shade determination

All groups showed a clinically visible change tooth shade as the ΔE_ab_ and ΔE_00_ values were higher than the 50:50% perceptibility (ΔE_ab_ = 1.2 and ΔE_00_ = 0.8) and 50:50% acceptability (ΔE_ab_ = 2.7 and ΔE_00_ = 1.8) thresholds. The mean and standard deviation values of ΔE_ab_ and ΔE_00_ are outlined in Table 4, whereas the mean and standard deviation values of ΔL, Δa, and Δb are described in Table 5.

**Table 4:** Mean and standard deviation values of ΔE_ab_ and ΔE_00_ between baseline (BL) and one‐month follow‐up.

	Mean (standard deviation)	
Groups	ΔE_ab_	ΔE_00_
PLACEBO	10.3 (3.1)^a^	5.2 (2.3)^a^
PBMT	11.2 (2.9)^a^	5.9 (1.9)^a^
CPPACPF	9.8 (3.0)^a^	4.7 (2.1)^a^
CPPACPF + PBMT	10.1 (3.0)^a^	4.9 (1.9)^a^

*Note*: Different lowercase letters represent significant difference in the intragroup evaluation, for the different tests applied; *p* ≤ .05.

Abbreviations: CPP‐ACPF, casein phosphopeptide‐amorphous calcium phosphate fluoride; PBMT, photobiomodulation therapy.

**Table 5:** Mean and standard deviation values of ΔL, Δa, and Δb between baseline (BL) and one‐month follow‐up.

	Mean (standard deviation)		
Groups	ΔL	Δa	Δb
PLACEBO	15.56 (2.5)^a^	2.3 (0.9)^B^	−6.15 (3.5)^c^
PBMT	14.91 (3.9)^a^	1.9 (1.2)^B^	−7.12 (2.1)^c^
CPPACPF	16.17 (2.2)^a^	1.7 (1.1)^B^	− 5.91 (2.3)^c^
CPPACPF + PBMT	15.43 (2.1)^a^	2.2 (0.7)^B^	−6.23 (2.9)^c^

*Note*: Different lowercase letters represent significant differences in the intragroup evaluation, for the different tests applied; *p* ≤ .05.

Abbreviations: CPP‐ACPF, casein phosphopeptide‐amorphous calcium phosphate fluoride; PBMT, photobiomodulation therapy.

## 4 DISCUSSION

In this study, the null hypothesis H0 was rejected as the desensitizing treatments tested were significantly different from each other in reducing TS after at‐home whitening. The PLACEBO group had the highest TS during the 21‐day bleaching treatment, and the VAS scores reported by the group gradually increased over the treatment, consistent with similar results from previous studies (Ontiveros et al., 2012; Rezende et al., 2019). The continuous use of the bleaching agent during at‐home whitening may have caused this gradual increase in painful symptoms. Furthermore, the timing of TS pain peaked at different time points between at‐home and in‐office whitening techniques. Generally, for in‐office whitening, pain peaks between 1 and 6 h after each TW session (Tay et al., 2009; Yassin & Milly, 2019).

The decrease in postwhitening TS with the application of A low‐intensity laser (PBMT) is often used to decreased related to post‐whitening TS postwhitening due to its analgesic and anti‐inflammatory effect (Alencar et al., 2018; de Paula et al., 2019; Lopes et al., 2017; Mayer‐Santos et al., 2017). This study found that the PBMT group had a significant decrease in TS compared with the PLACEBO group, which corroborates the findings of the meta‐analysis by Carneiro et al. (2022). This finding may be explained by the mechanism whereby PBMT reduces nerve conduction velocity in C fibers, which are responsible for transmitting painful stimuli. When the laser interacts with the tissue, the depolarization period increases, thus altering the pain threshold (Dilsiz et al., 2009; Terayama et al., 2020). Additionally, PBMT has an anti‐inflammatory effect that can suppress substance P and increase adenosine triphosphate and β‐endorphin release (Wakabayashi et al., 1993).

Although most scientific evidence supports using PBMT to reduce postwhitening TS, Calheiros et al. (2017) did not find significant differences in pain reduction between groups with and without PBMT (Calheiros et al., 2017). This variability in results could be explained by differences in the photobiomodulation parameters used across studies. In fact, of the major limitation of PBMT is the lack of a well‐established clinical protocol for TS reduction (Carneiro et al., 2022). The protocols available in the literature are highly diverse, and the success of PBMT depends on accurate dosimetry, thus requiring establishing the parameters that affect the treatment response, such as wavelength, energy density (J/cm^2^), energy per spot (J), total energy, number of irradiated spots, power (W), emission regime and specific irradiation characteristics (spot size diameter) (Moosavi et al., 2016).

The CPPACPF group also showed a significant reduction in TS over the 3‐weeks treatment period when compared to the PLACEBO group, in line with the results from previous studies (Alexandrino et al., 2017; Llena et al., 2019; Vasconcelos et al., 2012). The observed decrease in TS with the use of CPP‐ACPF paste may be explained by its remineralizing and obliterating effects (Berkathullah et al., 2018). CPP‐ACPF may reduce the penetration of peroxides into dental tissues, thereby reducing pulpal inflammation (de Vasconcelos et al., 2012; Reynolds, 1998). The CPP‐ACPF paste can be applied in‐office and at‐home. In this study, this paste was applied in office to standardize the application CPP‐ACPF and the decrease the risk of bias. When this paste is applied at home by the patient, the results also indicate a decrease in TS (Yassin & Milly, 2019).

Previous clinical trials have also investigatedcombining PBMT with desensitizing agents during a whitening treatment in the hope of inducing a synergistic effect on pain modulation (Moosavi et al., 2016; Yahya et al., 2022). However, a randomized clinical trial conducted by De Paula et al. (2019) evaluated the combination of a desensitizing agent based on 2% potassium nitrate with PBMT, but found no synergistic desensitizing effect. Nevertheless, the study revealed the rather promising effect of potassium nitrate or PBMT alone. Similar results were found by Pompeu et al. (2021) when combining 10% strontium chloride with PBMT, which was effective in reducing post‐whitening TS, but not significantly different from using PBMT or strontium chloride alone. In this study, combining PBMT with CPP‐ACPF proved more effective in reducing pain in the second and third weeks of evaluation than using PBMT or CPP‐ACPF alone. Furthermore, the CPPACPF + PBMT group showed a significant reduction in TS between treatment weeks in the intragroup analysis. The results suggest the effectiveness of combining PBMT with the CPP‐ACPF paste.

The synergistic effect resulting from combining the analgesic and anti‐inflammatory action of PBMT with the remineralizing potential of the CPP‐ACPF paste may have contributed to the improved reduction in post‐whitening TS. A previous study demonstrated that combining PBMT with CPP‐ACPF was effective in reducing dentin hypersensitivity (Guanipa Ortiz et al., 2019). The combination of these desensitizing treatments had not yet been tested for post‐whitening TS before the present study. Contrary to the sensitivity typically reported by patients who have exposed dentin, which is mainly related to thermal stimuli, bleached teeth can hurt even without any stimulus, thus indicating a different pain mechanism related to peroxides differs from the mechanism of other types of tooth pain (Markowitz, 2010; Markowitz & Pashley, 2008b).

In the present study, we used objective (spectrophotometric) methods to determine tooth shade. Spectrophotometric measurements provide greater accuracy than visual analysis using color scales they are are less susceptible to subjective judgments (Martins et al., 2020; Wetter et al., 2009). Moreover, we used the CIEDE2000 color‐difference formula and the CIELab color space system to evaluate color change. According to Sharma et al. (2005) the CIEDE2000 formula is most compatible with visual perception and acceptance of color change because shade measurements are fitted to three parameters: lightness, hue and chroma. Although, the advantages of CIEDE2000, the CIELab color space system remains commonly used to measure changes in shade during TW clinical trials (de Paula et al., 2019; Pompeu et al., 2021). Therefore, using the CIELab color space system enables to compare data between different clinical trials and may help to improve the scientific evidence.

The combination of CPP‐ACPF with PBMT did not affect the effectiveness of TW. The results of shade variation did not differ between the study groups, thus showing that none of the protocols used in this study negatively interfered with TW efficacy. Consequently, H02 cannot be rejected in this study. The clinical trials that have evaluated PBMT or CPP‐ACPF alone have also concluded that these desensitizing treatments did not interfere with the effectiveness of TW (Alencar et al., 2018; Alexandrino et al., 2017; de Paula et al., 2019; Gama Cunha et al., 2012; Pompeu et al., 2021). In this study, shade was determined in canines because they are the darkest teeth naturally, which allows more sensitive color change evaluation (Wetter et al., 2009). Rezende et al. (2016) observed that the teeth with the darkest shade at baseline undergo the strongest whitening process, consistent with previous studies in which whitening was greater in canines than in incisors, resulting in a harmonious bleaching outcome (Ontiveros et al., 2012; Wetter et al., 2009).

There are some limiting factors of this clinical trial that should be considered. Firstly, all participants were young adults with a mean age of 24 years, which may limit the generalizability of the results to the general population. However, only young patients were selected to prevent the physiological process of secondary dentin deposition, which decreases pulp volume and therefore innervation and vascularization, from introducing a bias to TS assessment after at‐home whitening (Abrams & Thompson, 2014; Farac et al., 2012; Melzack, 1975). Secondly, although VAS is a pain‐assessment instrument with validity and reliability reported in the literature, the subjectivity of self‐reported pain data may change the results because pain is aassessedbased on the individual's subjective perception of its intensity and, hence, depends not only on the physiological response but also on the emotional response (Melzack, 1975).

Future studies should investigate the efficacy of combining PBMT with CPP‐ACPF in controlling pain after in‐office whitening treatment. This is important because the intensity of TS directly increases with the concentration of bleaching agents, and in‐office whitening is associated with a higher intensity of TS compared with at‐home whitening. Although at‐home whitening was used in this study, a high concentration of carbamide peroxide (22%) was applied. Based on the method and results of this clinical trial, combining PBMT with CPP‐ACPF reduced post‐whitening TS without affecting with the effectiveness of the bleaching treatment. Moreover, from the second week of at‐home whitening, the combination of desensitizing treatments was more effective in reducing pain than using PBMT or CPP‐ACPF paste alone.

## 5 CONCLUSION

Therefore, based on this study data, PBMT associated with CPP‐ACPF proved be effective on treatment do TS posthome whitening, without compromising the efficacy of TW.

## AUTHOR CONTRIBUTIONS

Maria Eduarda Cardoso: Principal responsible for developing the clinical study and writing the manuscript. Clinical study operator. Administered desensitizing treatment to all patients treated. Elma Vieira and Raíssa Mesquita: assistants in all clinical appointments conducted in this study. They were responsible for laser blinding and patient guidance. Maria Eduarda Cardoso, Cristiane Alencar, and Elma Vieira: responsible for the design and definition of the statistical analysis of this work. Cecy Silva and Eliane Bemergui: responsible for the study design, coordinating all clinical phases of this research, and guiding and reviewing the manuscript production.

## CONFLICT OF INTEREST STATEMENT

The authors declare no conflict of interest.

## ETHICS STATEMENT

This study was submitted to the Human Research Ethics Committee of the Institute of Health Sciences at the Federal University of Pará (*Comitê de Ética em Pesquisa em Seres Humanos do Instituto de Ciências da Saúde da Universidade Federal do Pará* – CEP‐ICS/UFPA), approved under opinion number 49921321.2.0000.0018. This study was registered on the ClinicalTrials. gov website under the identifier NCT05298059. The participants were duly informed about the risks and methods of this study, and signed an informed consent form in accordance with the Declaration of Helsinki.

## Data Availability

Not applicable.
